# Births, Deaths and Marriages in England

**Published:** 1859-11

**Authors:** 


### *Births, Deaths and Marriages in England.*

The Register-General has issued his quarterly report of the marriages, births and deaths, registered in England during the last quarter. 84,090 persons married in the quarter that ended on June 30, or 4272 in excess of the numbers who married in the corresponding quarter of last year.—The births of 168,611 children were registered in the quarter that ended on September 30. The number is 10,862 in excess of the births of the corresponding quarter of last year. 63,972 was the excess of the number of births over the number of deaths, and that was, therefore, the natnral increase of the population of England and Wales in 92

days. On an average, 695 were added to the population daily, and the probable daily increase of the population of the United Kingdom, was 1042, which, at the ordinary rates of mortality, will supply 347 men daily, of the age of 20. 104,330 persons died in the last quarter. This number is 6079 in excess of the deaths, 98,260, in the corresponding quarter of last year.

